# How is the acyl chain composition of phosphoinositides created and does it matter?

**DOI:** 10.1042/BST20190205

**Published:** 2019-10-28

**Authors:** David Barneda, Sabina Cosulich, Len Stephens, Phillip Hawkins

**Affiliations:** 1 Oncology R&D, AstraZeneca, Cambridge, U.K.; 2 Signalling Programme, Babraham Institute, Cambridge, U.K.

**Keywords:** lipid metabolism, lipid-binding domains, membranes, phosphatidylinositol, polyunsaturated fatty acids, signalling

## Abstract

The phosphoinositide (PIPn) family of signalling phospholipids are central regulators in membrane cell biology. Their varied functions are based on the phosphorylation pattern of their inositol ring, which can be recognized by selective binding domains in their effector proteins and be modified by a series of specific PIPn kinases and phosphatases, which control their interconversion in a spatial and temporal manner. Yet, a unique feature of PIPns remains largely unexplored: their unusually uniform acyl chain composition. Indeed, while most phospholipids present a range of molecular species comprising acyl chains of diverse length and saturation, PIPns in several organisms and tissues show the predominance of a single hydrophobic backbone, which in mammals is composed of arachidonoyl and stearoyl chains. Despite evolution having favoured this specific PIPn configuration, little is known regarding the mechanisms and functions behind it. In this review, we explore the metabolic pathways that could control the acyl chain composition of PIPns as well as the potential roles of this selective enrichment. While our understanding of this phenomenon has been constrained largely by the technical limitations in the methods traditionally employed in the PIPn field, we believe that the latest developments in PIPn analysis should shed light onto this old question.

## Introduction

**Figure 1.**
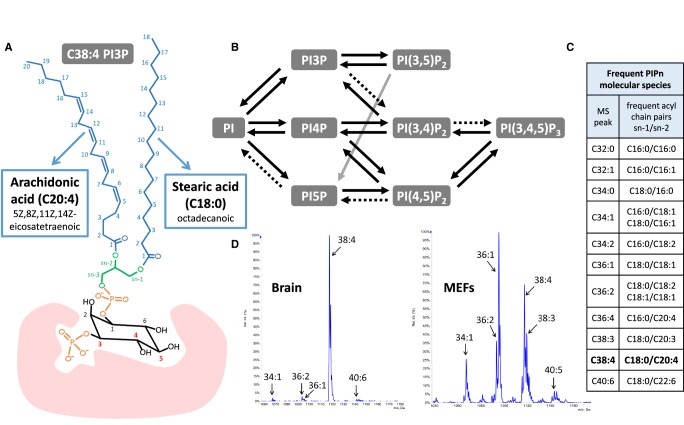
Phosphoinositide structures, metabolism and interactions with effectors. (**A**) A typical phosphoinositide (PIPn) structure is represented by C38:4 PI3P (sn-1-stearoyl-2-arachidonoyl phosphatidylinositol 3-phosphate). The fatty acids acylated in the sn-1 and sn-2 positions of the glycerol backbone (green) are labelled with both their common and systematic name, and with the lipid nomenclature based on the number of carbons and double bonds in brackets. The three positions in the inositol ring which can be phosphorylated or dephosphorylated by specific kinases and phosphatases to give rise to the different members of the PIPn family are indicated by red numbers. The pink cartoon engulfing the phosphorylated ring represents a specific interaction between the phosphorylated inositol ring and the binding domain of a PIPn effector protein. (**B**) Pathways of PIPn metabolism indicating their dynamic interconversion by kinase and phosphatase reactions acting on different positions on their inositol rings. Disputed activities are indicated with dashed arrows. (**C**) List of PIPn molecular species frequently identified by MS analysis. When their acyl chain composition is not resolved by further fragmentation, they are expressed by the total number of carbons and double bonds in both chains. (**D**) MS profile of the PIP_2_ molecular species found in mouse brain and mouse embryonic fibroblasts (MEFs) (adapted from [123]). The three PIP_2_ isomers are not resolved by this method, though PI(4,5)P_2_ generally accounts for most of the signal. While brain PIP_2_ shows the predominance of C38:4 species characteristic of mammalian PI, this enrichment can be lost in cultured cells such as MEFs, which present an heterogeneous distribution of molecular species in all PIPn.

Phosphoinositides (PIPn) are a family of eight phospholipids (PI, PI3P, PI4P, PI5P, PI(4,5)P_2_, PI(3,4)P_2_, PI(3,5)P_2_, PI(3,4,5)P_3_), whose members define membrane identity, drive compartment-specific accumulation of other lipids and transduce extracellular signals [1]. They are interconverted by a series of highly regulated PIPn kinases and phosphatases and a key principle by which they act in cells is the specific recognition of their phosphorylated head-groups by enzymes and effector proteins [2] (Figure 1). Where known, this recognition is mediated primarily through electrostatic interactions between inositol ring phosphates and amino acid side chains in the conserved pockets of target proteins with less clear specific interactions with the hydrophobic ‘backbone’. Nonetheless, an under-appreciated characteristic of PIPn in mammalian cells is that they often display a relatively molecularly homogeneous acyl chain composition, the mechanistic significance of which is unclear.

### PIPn in mammalian tissues is enriched in the C38:4 molecular species

Glycerophospholipid classes are defined by their polar head-groups, which are bound to a diacylglycerol (DG) backbone through a diester phosphate. Each of these classes comprise a range of molecular species, which share the same head-group, but present different pairs of aliphatic chains (Figure 1). These chains can be linked via ester or ether bonds and derived from fatty acids of different length and degree of saturation [3]. Most phospholipid classes present a wide variety of alkyl/acyl compositions and only occasionally is the function of a particular molecular species known e.g. ‘rigid’ C32:0-PC dominates lung surfactant [4]. However, the acyl chain pairings of mammalian PI are unusually enriched (≥70%) in a single molecular species, composed of a stearoyl chain in sn-1 (18 carbons and no double bonds; C18:0) and an arachidonoyl chain in sn-2 (20 carbons and 4 double bonds; C20:4) (Figure 1) [5–7]. As both chains are simultaneously measured in most routine, mass spectrometry (MS)-based analyses, this molecular species is usually referred to as ‘C38:4’ PI. Early analyses focussed only on PI, but recent MS studies have shown that the acyl compositions of PI, PIP, PIP_2_ and PIP_3_ are very similar (current MS methods do not discriminate between regio-isomers), consistent with rapid interconversion between the various phosphorylated forms of these lipids and parental PI (Figure 1) [8,9]. These observations suggest evolution has favoured the selective enrichment of this species of PIPn in mammals, but we know very little about the advantages provided by this acyl chain species and the molecular mechanisms responsible for creating it.

## How is the acyl composition of mammalian PIPn determined?

**Figure 2.**
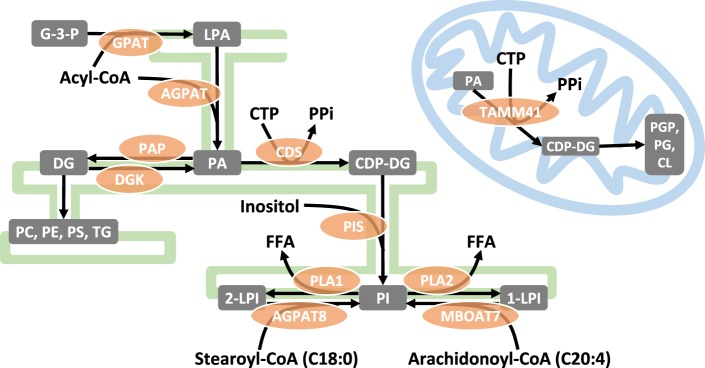
Synthesis of phosphatidylinositol and acyl chain remodelling. De novo synthesis of PI starts with the formation of the simple phospholipid PA in the ER. At this point, glycerophospholipid synthesis diverges into two branches: the CDP-DG pathway for the synthesis of PI and the DG pathway for the other ER lipids. CDP-DG is also the precursor of CL but its synthesis takes place in the mitochondria using a different CDP-DG synthase (TAMM41). A selective channelling of C38:4 PA molecules towards the CDP-DG branch might contribute to the enrichment of this species in PI. Once synthesized, PI molecules can be remodelled into C38:4 PI by the Lands cycle, which involves the release of one of their acyl chains and the re-acylation of the resulting LPI via acyltransferases selectively incorporating C18:0 and C20:4 chains in sn-1 and sn-2, respectively. Abbreviations: ER, endoplasmic reticulum; G-3-P, glycerol-3-phosphate; LPA, lysophosphatidic acid; PA, phosphatidic acid; DG, diacylglycerol; PC, phosphatidylcholine; PE, phosphatidylethanolamine; PS, phosphatidylserine; TG, triacylglycerol; PGP, phosphatidylglycerol phosphate; CL, cardiolipin; CDP-DG, CDP-diacylglycerol; PI, phosphatidylinositol; LPI, lysophosphoinositol; FFA, free fatty acid; CTP, cytidine triphosphate; PPi, pyrophosphate; GPAT, glycerol-3-phosphate acyltransferase; AGPAT, acylglycerol-3-phosphate acyltransferase; PAP, phosphatidate phosphatase; DGK, diacylglycerol kinase; CDS, CDP-diacylglycerol synthase; TAMM41, TAM41 mitochondrial translocator assembly and maintenance homologue; PIS, phosphatidylinositol synthase; PLA1/2, phospholipases A1/A2; AGPAT8, 2-acylglycerol-3-phosphate acyltransferase 8; MBOAT7, membrane bound O-acyltransferase 7.

In principle, the C38:4 species of PI could be selectively synthesized *de novo* and/or enriched by subsequent acyl chain ‘remodelling’. The pathways for *de novo* synthesis of PI and acyl chain remodelling by the ‘Lands Cycle’ (Figure 2) have been discussed recently in an excellent review [10]; these reactions are envisaged to occur in the ER or membrane domains closely associated with it. Attempts to understand the major points of C38:4 enrichment have employed measurements of the acyl chain compositions of key intermediates, radioactive tracer studies and interrogation of the acyl chain selectivity of relevant enzymes *in vitro*. However, due to both technical and interpretational difficulties, the picture is still far from clear.

### C38:4 enrichment by *de novo* synthesis

Phospholipid synthesis branches in the ER from the common precursor PA by the action of PA phosphatases, generating DG for PC/PE/PS/TG synthesis or, the action of CDS1/2 generating CDP-DG for PI synthesis [10] (Figure 2). PGP/PG/CL synthesis in mitochondria also uses a CDP-DG intermediate but this seems to be mostly synthesized *in situ* by TAMM41, a CDP-DG synthase that evolved independently of the ER enzymes, CDS1/2 [11,12]. As most CDP-DG in the ER will be committed to PI synthesis, a possible enrichment mechanism could be the selective channelling of C38:4 PA towards the CDP-DG branch and non-C38:4 PAs towards the DG branch (Figure 2). Indeed, the activation of PA phosphatases in ER domains proximal to the nuclear envelope represses PI synthesis in this region [13], but it is not known if this process is selective for certain molecular species. Most studies have reported the presence of multiple molecular species of PA in cells [14], but there are very few measurements of the acyl chain composition of CDP-DG (mainly due to its chemical and biological instability) [15]. Furthermore, given CDP-DG is used for both PI and PGP synthesis, ER and mitochondrial pools would need to be distinguished to obtain unambiguous evidence of C38:4 enrichment in the CDP-DG destined for PI synthesis. Some evidence suggesting selective channelling of PA species towards CDP-DG comes from investigations of the acyl chain selectivity of CDS1 and 2 *in vitro*. These studies suggest CDS2 has a significant preference for C20:4 in the sn-2 position of PA, but CDS1 is less selective [16], although it prefers unsaturated species of PA compared with fully saturated species [17]. CDS2 is ubiquitously expressed, while CDS1 is restricted to a subset of tissues [16,18] and might be induced when a high rate of *de novo* PI synthesis is required [19]. Furthermore, overexpressed CDS1 and CDS2 show slightly different patterns of localization in the ER [17]. These observations suggest that the formation of CDP-DG from PA is likely to be a regulated step and CDS1 and 2 have evolved to play differing roles, although definitive evidence for their involvement in establishing the acyl chain composition of CDP-DGs is still lacking.

There is only one PIS activity in mammalian cells [10]. This enzyme does not possess relevant acyl chain selectivity *in vitro* [20] and there are several examples where mammalian cells can synthesise a range of different PI species in culture (see below). This suggests PIS will use any CDP-DGs available to it and is very unlikely to select C38:4 CDP-DG for PI synthesis.

Early metabolic labelling studies comparing the incorporation rate of ^14^C-glycerol, ^14^C-glucose, and ^32^P-Pi showed that the initial stages of *de novo* PI synthesis produce multiple PI species, which are then followed by a progressive enrichment of the C38:4 species [21–24]. This is the best evidence to date that the *de novo* synthesis pathway does not selectively produce C38:4 PI, but that this species is enriched by subsequent steps. It is therefore surprising that, apart from one preliminary report [25], these pioneering studies have not yet been confirmed and extended in any detail with modern cell culture and MS methods.

### C38:4 enrichment by acyl chain remodelling

The foregoing discussion suggests at least some, if not all, of the C38:4 enrichment in PIs is achieved by acyl chain remodelling of multiple PIs synthesized *de novo*. The primary mechanism for engineering new acyl chain variants of pre-existing phospholipids is the Lands Cycle. In this cycle, a phospholipase A (PLA) hydrolyzes an acyl chain from the sn-1 or sn-2 position and the resulting lysophospholipid is reacylated with a new acyl chain by a lysophospholipid acyltransferase (Figure 2). In principle, the process of acyl chain enrichment could be governed by the acyl chain selectivity of phospholipases A1/2 or the acyl-CoA transferases, together with the availability of different acyl-CoAs. Thus far, the clearest examples of selectivity are found amongst the large family of acyl-CoA transferases, some of which have strong preferences for a particular combination of lyso-lipid and acyl-CoA [26].

MBOAT7 (Membrane-bound O-acyltransferase 7) can selectively reacylate LPI in sn-2 with polyunsaturated substrates, such as arachidonoyl-coA [27,28], while AGPAT8 (1-acylglycerol-3-phosphate acyltransferase 8), can reacylate LPI in sn-1 using stearoyl-coA [29]. Despite initial studies suggesting that AGPAT8 can acylate lysocardiolipin and other lysophospholipids *in vitro* [30–32], the main effect of AGPAT8 deletion in cells and animals is a decrease in the stearoyl content of PIPn [7,29,33], suggesting that its main function is the sn-1 remodelling of PI. The extent to which MBOAT7 and AGPAT8 contribute to C38:4 enrichment in PI by selectively incorporating arachidonoyl and stearoyl chains in sn-2 and sn-1, respectively, is shown by the reduction in C38:4 PIPn species in both MBOAT7−/− [5,6] and AGPAT8−/− [7] mice. In both cases, the reduced frequency of C38:4 species was accompanied by a decrease in PI levels, possibly due to the impairment of LPI reacylation, though it could also indicate that the C38:4 backbone is involved in the maintenance of the total PIPn pool. Nevertheless, despite the relative increase in other species, C38:4 was still predominant in both KO models, indicating that other mechanisms must also contribute to its enrichment.

AGPAT8−/− mice appeared healthy, despite the decrease in PI relative to other lipids and a reduced proportion of C18:0 in PIPn [7]. However, its transient silencing in ARPE-19 cells, derived from human retinal pigment epithelium, which also reduced the C18:0 content in PIPn, selectively altered the levels and localization of PI3P and PI(4,5)P_2_, hence affecting membrane trafficking dependent on these lipids [33]. The mechanisms behind these alterations remain unknown, though it has been suggested that the substitution of the C18:0 chain in sn-1 for the shorter C16:0 could affect PIPn interaction with proteins sensitive to the orientation or distance of the polar head with respect to the membrane surface [34].

MBOAT7−/− mice displayed neonatal lethality and aberrant brain development, but it is not clear whether these anomalies are caused by the accumulation of LPI and a decrease in the total levels of PIPn rather than the modest changes in their distribution of molecular species [5,6]. The rs641738 C > T variant in the locus containing MBOAT7 has been associated with liver diseases such as alcohol-related cirrhosis [35], inflammation and fibrosis in chronic hepatitis B and C [36,37] and non-alcoholic fatty liver disease, though this later association has been disputed [38]. This variant is associated with lower protein levels of MBOAT7 in liver and changes in the levels of minor PI species in plasma [39] and liver [40]. Nevertheless, it seems likely that this pro-inflammatory phenotype may be related to defective processing of C20:4 by MBOAT7 rather than small changes in PI species.

The first committed step in PI remodelling is its hydrolysis into LPI by PLAs, and thus coupling this process to *de novo* PI synthesis might be envisaged to facilitate remodelling. Indeed, it has been suggested that the faster remodelling of atypical PE and PS species produced *de novo* involves their selective degradation by PLAs [41]. Similarly, PIs generated from exogenous C32:0 CDP-DG in liver microsomes were rapidly hydrolyzed into LPI, while little LPI was formed from pre-existing PIs, suggesting a selective de-acylation of newly synthesized PIs [42]. As yet, no PI-PLA activity selective for these intermediate species has been identified and the roles of the PLA superfamily of enzymes in phospholipid remodelling are not clearly defined [43]. The inhibition of Ca^2+^-independent phospholipase A2b (iPLA2b) reduces the incorporation of C20:4 into phospholipids [44,45] while its overexpression increases the levels of C38:4 PI [46]. The analysis of brains from iPLA2b−/− mice, however, suggest that it is not required for C38:4 enrichment [47]. DDHD1 seems to be the PLA1 involved in the generation of C20:4 LPI [48], an endogenous activating ligand of G protein-coupled receptor GPR55 [49,50]. Although loss of the homologue of DDHD1 in *C. elegans*, IPLA-1, results in a dramatic change in their predominant PI species, from C18:0/20:5 to 18:1/20:5 [29], in DDHD1−/− mice there were no changes in C38:4 PIPn whilst there was a relative increase in 18:1/C20:4 PIPn at the expense of shorter and more saturated species [51]. Regarding the products of PLA activity on PIs, C18:0 LPI shows the highest levels in mouse tissues, with C20:4 LPI also being abundant in the nervous system [52]. However, this provides little information about the kinetics of remodelling in sn-1 and sn-2 as these species can be formed as remodelling intermediates but also from C38:4 PI for signalling purposes. Thus, if it is not reacylated, LPI can be metabolized by lysophospholipases A, C or D to generate glycerophosphoinositol [53], monoacylglycerol [54] or LPA [55], which will all have different signalling roles [56].

In addition to the acyl-coA acyltransferases, PI acyl chains might also be remodelled by poorly characterized transacylation reactions, in which an acyl chain is transferred directly from a phospholipid donor to a lysophospholipid [57]. This may be an important reaction for CL remodelling, as evidenced by the mitochondrial dysfunction caused by defects in the CL transacylase Tafazzin [58], but the potential for this type of reaction in PI remodelling remains unknown.

Selective remodelling of *de novo* PI species could also be facilitated by a spatial confinement of the enzymes involved in PI synthesis and remodelling. Regarding this possibility, a growing body of evidence based on the localization of overexpressed PIS indicates that PI synthesis occurs in discrete locations in the ER [59–62]. PI synthesis and remodelling could be coupled in these domains, as indicated by the co-localization AGPAT8 with PIS [33].

In summary, we still do not fully understand the process of acyl chain enrichment for the C38:4 species of PI in mammalian cells. It remains possible that some selectivity occurs through the actions of CDS2 to supply C38:4-enriched CDP-DG to PIS, but most evidence points to a significant role for acyl chain remodelling of PIs synthesized *de novo* by the LPI acyltransferases MBOAT7 and AGPAT8, and possibly the PLA1 DDHD1. However, the deletion of these acyl transferases in mice does not affect the relative levels of C38:4 in PIPn to the same extent as it does in *C. elegans*, suggesting redundant mechanisms are co-ordinated in mammals to ensure a robust C38:4 enrichment. Further studies are clearly needed to establish the mechanisms controlling this efficient remodelling of PIs, including its compartimentalization and its coordination with PI synthesis.

## What are the selective properties of the acyl chain composition of PIP(n)?

The important properties conveyed by C38:4 enrichment in mammalian cells are still unclear. Plausible arguments can be made in favour of a creating a distinct molecular label to distinguish the PIPn backbone from other lipids, a biophysical advantage to the mechanisms by which PIPn interact with their protein effectors or, a regulated supply of arachidonic acid for the synthesis of bioactive lipids.

### Does C38:4 enrichment create an efficient ‘PI’ cycle?

**Figure 3.**
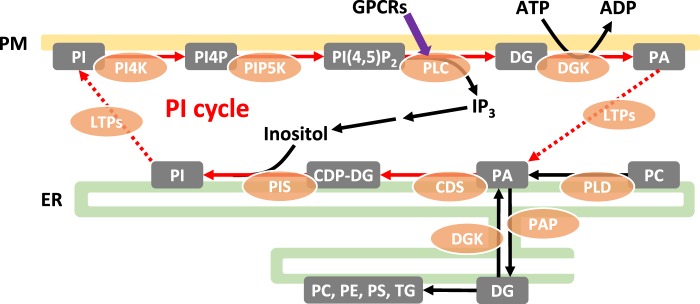
The signal PLC transduction pathway is coupled to PI re-synthesis to maintain the PIPn pool. The activation of PLC (phospholipase C) in response to GPCR agonists (G protein-coupled receptors) induces the cleavage of PI(4,5)P_2_, generating DG (diacylglycerol) and IP_3_ (inositol 1,4,5-trisphosphate), which leads to the release of Ca^2+^ from intracellular stores and the activation of PKC (protein kinase C), respectively. The drop in the total PIPn pool caused by PI(4,5)P_2_ hydrolysis is compensated by the activation of PI synthesis. It has been proposed that this PI re-synthesis involves a closed ‘PI cycle’ (red arrows), where the DG generated by the hydrolysis of PI(4,5)P_2_ is selectively recycled back into PI to maintain the total PIPn pool. This process involves the transport of the generated DG and/or PA from the PM (plasma membrane) to the ER (endoplasmic reticulum), where it can be utilized by PI synthesis enzymes CDS and PIS. This cycle could be spatially confined to PM-ER contact sites, where LTPs (lipid transfer proteins) transport the lipid intermediates between membranes (dotted lines). The C38:4 backbone could facilitate the PI cycle by providing molecular identity with the DG and PA molecules, preventing their consumption by other biosynthetic pathways as well as the utilization of intermediates from other origins, such as PA generated from PC via PLD (phospholipase D).

The field of PIPn signalling originated from the discovery of a hormone-stimulated increase in the metabolism of PI relative to other phospholipids. Some of these early studies described a selective increase in the incorporation of ^32^P-Pi or ^3^H-inositol compared with ^14^C-glycerol, which was interpreted to mean a relatively ‘closed’ PI cycle may exist in which a stimulated PLC produces DG and PA that are selectively recycled back into PI (Figure 3; [63]). The activation of a PI(4,5)P_2_-selective PLC by receptors is now accepted to be a central signal transduction mechanism in multicellular organisms and liberates the ‘second messengers’ IP_3_ and DG, which stimulate intracellular Ca^2+^-release and the activation of PKC, respectively [64,65]. Whilst PIPn can be interconverted by kinases and phosphomonoesterases, the action of a PLC produces DG, which is a common biosynthetic intermediate in lipid metabolism. This creates a potential problem for cells in segregating PLC-derived-DG from other sources of DG, enabling it to both act as a selective signal for the activation of PKC and also as a source of PA for the PI-cycle. Furthermore, PLC activation is often observed to occur in parallel with activation of a PLD which usually appears to be directed predominantly against PC, adding a further source of complexity in segregating pools of PA (and DG via PA phosphatases) destined for different purposes [66].

Recent studies have suggested an important element in the organisation of a ‘PI cycle’ that may allow segregation from other biosynthetic pathways is the close and discrete apposition of contact sites between the ER and PM (Figure 3) [67–69]. These ER-PM contact sites offer an opportunity for lipid transfer proteins (LTPs) to locally exchange PLC-derived DG and PA produced in the PM, with newly synthesized PI produced in the ER, offering the potential for close coupling of PI-resynthesis and -consumption and the possibility of a diffusion barrier with bulk lipid synthesis in more distant regions of the ER [70–73]. There are still many details missing however, as to the precise composition of different types of ER-PM contact sites and how effectively they are isolated from other regions of the ER.

It seems conceivable that the spatial segregation offered by ER-PM contact sites might be supplemented in mammals by molecular recognition of the C38:4 backbone. Thus, the common C38:4 signature in intermediates generated from PI(4,5)P_2_ hydrolysis could influence their selective recycling back into PI. A preference for C38:4 substrates has been observed with CDS2 [17] and other PI cycle enzymes, such as DGKε [74] and PI4P5Ks [75], and an interesting idea has been proposed that the PI cycle could act as a ‘molecular sieve’, progressively enriching the C38:4 species through multiple passages around the cycle [76]. However, the relevance of this selectivity for C38:4 substrates is still unclear. DGKε−/− mice do not present major anomalies, but they do display decreased levels of C20:4-containing PIP_2_ in the brain and an apparent attenuation of PLC signalling induced by electroconvulsive shock [77]. PLC signalling was also affected by the deletion of MBOAT7 in the RAW264.7 macrophage cell line, which caused a significant reduction in the % of C38:4 in PIPn that was accompanied by prolonged Ca^2+^ oscillations upon stimulation, though it is not clear if the apparent reduction in Ca^2+^ efflux could be linked to a defective PI cycle [78].

### Does C38:4 enrichment affect interactions with effectors?

**Figure 4.**
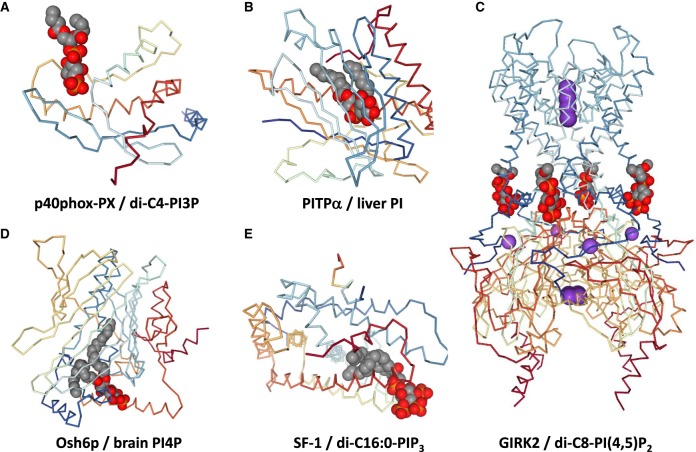
Phosphoinositide–protein interaction modes. The influence of the acyl chains in PIPn–protein interactions is often missed in structural studies by the utilization of soluble, short-chain versions of PIPn, the conformational dynamics of longer chains and the lack of a membrane context. (**A**–**E**) Examples of protein–PIPn interactions adapted from the Protein Data Bank, where the protein backbone is shown interacting with PIPn of diverse acyl chain composition (in spacefill representation: carbon, grey; phosphorus, orange; oxygen, red). The original lipid ligand is indicated, although shorter versions are represented in the structures of PITPα (**B**) and GIRK2 (**C**) where the PIPn acyl chains were not fully solved. Although the interaction with PIPn-binding domains is often restricted to the phosphorylated inositol ring (**A**), its presentation relative to the membrane surface could be affected by the acyl chain length/saturation. The PIPn acyl chains can directly interact with hydrophobic residues inserted in the membrane in transmembrane proteins such as GIRK2 (**C**). Interestingly, the activation of certain members of this family of ion channels by PIPn was largely dependent of their acyl chains [80]. The influence of the acyl chains could be more evident in PIPn–protein interactions that involve the extraction of the whole lipid from the membrane (**B**,**D**,**E**). In these proteins, the polar head can be buried in the protein (**B**), with the acyl chains occupying two hydrophobic channels, but can also be exposed to the solvent (**E**), which could be indicative of a major binding contribution for the acyl chains. (**A**) PX domain from p40phox bound to di-C4-PI3P (PDB 1H6H) [124]. (**B**) PITPα complexed to PI from bovine liver (PDB 1UW5) [125]. (**C**) G protein-gated inward rectifier K+ channel GIRK2 in complex with sodium and di-C8-PI(4,5)P_2_ (PDB 3SYA) [126]. (**D**) Osh6p in complex with brain PI4P (4PH7) [127]. (**E**) nuclear receptor SF-1 bound to di-C16:0-PIP3 (PDB 4QJR) [92].

Many of the well-established interactions between PIPn and their effector proteins take place through the recognition of phosphorylated inositol head-groups by conserved domains in soluble, or extrinsic membrane proteins [2]. These interactions can induce a conformational change in the target protein and/or promote a significant change in its cellular location, causing an increase in effective concentration at the membrane where the particular PIPn resides [2]. Coupled with tight control of the synthesis and degradation of individual PIPn, these types of interactions allow PIPns to regulate compartment-specific events, for example the synthesis of PI(3,4,5)P_3_ at the plasma membrane regulates signal transduction through PH-domain containing effectors in response to growth factors, or synthesis of PI3P in early endosomes regulates protein trafficking through PX and FYVE domain-containing effectors. Several structures are now available for these complexes, which suggest the key interactions are electrostatic in nature and are confined to the inositol ring (an example of a PX domain bound to PI3P is shown in Figure 4A). However, many of these structures were obtained from crystals containing only a soluble inositol phosphate head-group or short-chain versions of PIPn, precluding the discovery of important interactions with the acyl chains. Furthermore, when specifically interrogated in biochemical assays, some of these interactions have been shown to be more efficient with C38:4 PIPn e.g. in the PDK1-mediated phosphorylation of PKB stimulated by PIP_3_ [79].

PIPn have also been found to interact specifically with a small number of intrinsic membrane proteins, such as members of the family of inward-rectifier potassium channels (IRKs) [1]; an example of PI(4,5)P_2_ bound to GIRK2 is shown in Figure 4C. Although the positions of the acyl chains are undefined in these structures (presumably because of their mobility), there is other evidence that the acyl chain composition is indeed relevant to these types of interactions. Thus, while constitutively active IRK1 selectively interacted with PI(4,5)P_2_ irrespective of its acyl chain composition, the G-protein activated GIRK1 and GIRK4 were promiscuously stimulated by PI(4,5)P_2_, PI(3,4)P_2_, PI(3,5)P_2_ or PIP_3_, but showed a strong preference for C38:4 over C32:0 species [80].

In principle, protein interactions involving the whole PIPn molecule, rather than just the head-group, might be more sensitive to acyl chain composition. This would be the case for LTPs, some of which can selectively extract individual phospholipid molecules from a donor membrane and transfer them to an acceptor membrane *in vitro* [68,81]. It is now clear that many LTPs are involved in non-vesicular transport of lipids between cellular membranes, though precisely how they achieve this is still under some debate [82]. An intriguing recent model suggests some members of the ORP/Osh family facilitate counter transport of distinct lipids, the direction of net transport of one lipid being created by the concentration gradient of a ‘driver’ lipid, PI4P, which is, in turn, determined by the separate locations of PI 4-kinases (on the acceptor membrane) and PI4P-4-phosphatases (on the ‘donor’ membrane), e.g. in PI4P-driven transport of PS between the ER and PM or, PI4P-driven transport of sterol between the ER and Golgi [83]. The crystal structures of several LTPs are known and they present various topologies for lipid binding, but often possess a hydrophobic groove or channel which can accommodate the acyl chains of phospholipids and separate them from the aqueous environment [81]; an example of PITPα bound to PI is shown in Figure 4B and Osh6p bound to PI4P is shown in Figure 4D. However, there is still very little data generated on the acyl chain selectivity of these LTPs for their cargo and the precise positions of the acyl chains in the available structures are frustratingly inexact, through a combination of acyl chain mobility and use of heterogenous lipid ligands (see Figure 4). The LTPs ORP8 and E-SYT2 expressed in mammalian cell lines co-purified with multiple phospholipid species [84,85] and other work has indicated that ORP8 can selectively interact with different acyl chain species of PI4P (and PI(4,5)P_2_) and suggests it might preferentially extract saturated species of PI4P from the plasma membrane [86]. Recent work has also suggested that the ORP homologues in yeast which regulate the transport of PS and cholesterol from the ER to the PM in exchange for PI4P, allow the creation of PM lipid nanodomains where sterol/PS aggregate with PI4P molecules remaining in the PM and facilitate their phosphorylation by PI4P5K. Remarkably, these sterol/PS/PI4P nanodomains were more efficiently established with unsaturated versions of PS and PI4P, and promoted the activation of PI4P5K by engaging with its amphipathic specificity loop [87]. Furthermore, whilst an analysis of PIs bound to PITPNC1 suggested a preference for C38:4 PI species, while shorter and more saturated species were bound to PITPα [88], there has been no systematic analysis of the acyl chain specificities of LTPs thought to be relevant to the counter exchange of DG, PA and PI across ER-PM contact sites, such as Nir2/3 and TMEM24. This would represent an obvious point at which selectivity for the C38:4 backbone might contribute to an effective PI cycle (see above).

It is also interesting to consider PIPn–protein interactions in the nucleus. Although PIPn are involved in the regulation of intra-nuclear processes, such as transcription or DNA-repair, their biophysical state in such a hydrophilic environment remains unclear, and little is known about their origin and acyl chain composition [89,90]. Small structures enriched in PI(4,5)P_2_ named Nuclear Lipid Islets have been immunoprecipitated and subjected to lipid analysis [91], but the distribution of PIPn molecular species has not been described. Crystal structures of PI(4,5)P_2_ and PIP_3_ bound to nuclear receptor SF-1 (Steroidogenic Factor-1) revealed that this interaction takes place through their hydrophobic tails (see Figure 4E), leaving the phosphorylated head exposed to the surface, where it could modulate co-activator binding [92] and be modified by PIPn kinases/phosphatases [93]. It is not known if this binding is affected by the acyl chain composition of the PIPn, though C32:0 species were used to derive the SF-1 crystals (Figure 4E). It has also been described that nuclear p53 can bind PI(4,5)P_2_, which results in its stabilization [94]. This interaction was resistant to denaturation, and was observed with natural PI(4,5)P_2_ but not with dioctanoyl-PI(4,5)P_2_.

### Lessons from evolution

The synthesis of PI from CDP-DG and free inositol via PIS is conserved in all eukaryotes (reviewed in [95]), which also present most of their phosphorylated forms [96]. While these PIPn participate in homologous functions in different organisms, the PI enrichment in C38:4 backbones has only been observed thus far in vertebrates. Therefore, tracing the emergence of C38:4 enrichment during the evolution of PIPn signalling could provide some insights into the evolutionary pressures favouring a distinct and homogeneous acyl chain composition in this key family of signalling lipids.

While C38:4 enrichment might be restricted to vertebrates, a common feature of PIPn across many different organisms is the concentration of saturated acyl chains in sn-1 compared with other phospholipids [29,97,98]. In yeast, the enrichment in C16:0 or C18:0 in sn-1 appears to take place by distinct mechanisms; the PI synthase Pis1p seems to select CDP-DG molecules containing C16:0 [99], while C18:0 chains are incorporated via remodelling by the LPI acyltransferase Psi1p, their closest homologue to mammalian AGPAT8 [100]. *Psi1Δ* yeast strains present an abnormal localization of PI4P and PI(4,5)P_2_ and alterations in vesicle trafficking and cell polarity, which has been attributed to non-redundant functions of the PIPn pool containing C18:0 [101]. The main lysophospholipid sn-2 acyltransferase in yeast microsomes is Ale1p, an MBOAT family enzyme with a strong preference for mono- and poly-unsaturated acyl-CoA chains, though the longer chain C20:4-CoA is a poor substrate [102]. Studies investigating the substrate preferences of the mammalian MBOAT family suggest some overlapping selectivity for the lyso-phospholipid head group and varying selectivity for acyl-CoA chain length and degree of unsaturation [26], but MBOAT7 appears remarkably selective for both LPI and C20:4-CoA [28]. Similar results are observed *in vitro* with homologues in nematode [27] and fly [103], though C20:4 is absent from flies [104]. Therefore, during the evolution of PIPn signalling, two different families of acyltransferases (AGPAT and MBOAT) have evolved to generate a pool of PIPn with a stearoyl chain in sn-1 and a polyunsaturated fatty acid in sn-2, which in vertebrates becomes C20:4. Remarkably, the social amoeba *Dictyostelium discoideum*, which branched before the divergence of fungi and metazoa, also presents a unique hydrocarbon backbone in PIPn, composed of an ether-linked C16:0 chain in sn-1 and a ester-linked C18:1 chain in sn-2 (C34:1e) [105]. Taken together, a theme emerges that suggests a core functional advantage to the selected PI backbone, such as availability within the bilayer and/or IPn head-group presentation to effectors. This advantage may be fine-tuned in individual organisms to the precise temperature and lipid composition of the membranes in which they must act. Moreover, in some organisms, such as vertebrates and *Dictyostelium*, there appears to have been further enrichment to more precise molecular species, which may imply additional advantages, such as metabolic identity and optimal PIPn–protein interactions.

While the hydrolysis of PI(4,5)P_2_ by PLC seems to be ubiquitous in eukaryotes, the emergence of PLC signalling in metazoans might have increased the pressure for PIPn homeostatic mechanisms. In fly photoreceptor cells, which present heterogeneous PIPn species and require the rapid replenishment of PI(4,5)P_2_ to sustain their light-activated PLC signalling, an efficient PI cycle is organised by a specialized ER-PM contact site, the Submicrovillar Cisternae, where PI is resynthesized using PA molecules transferred from the PM by RdgB proteins (a homologue of mammalian Nir2) [68]. In mammals, the efficient operation of a PI cycle in different cell types might be facilitated by an additional layer of selectivity provided by the C38:4 backbone (see discussion above). This may be reflected in the divergence of C38:4-selective CDS2 and non-selective CDS1 in vertebrates [106] and it would be informative to investigate whether other proteins involved in the mammalian PI cycle, such as Nir2/3, have evolved selectivity for C38:4 substrates.

## The presence of alternative PIPn species

Whilst the forgoing discussion has focussed on the enrichment of the C38:4 species of PIPn in primary mammalian tissue, several examples have been reported where this enrichment does not occur, or at least not to the same extent [107]. For instance, PI species in mouse testis and platelets present a relatively high proportion of palmitoyl (C16:0) chains [7,108]. High levels of a rare CL species composed by C16:0 chains have also been reported in testis, suggesting that these saturated phospholipids could have specific roles during spermatogenesis [109]. Regarding platelets, C32:0 is moderately abundant in PI but not in PIP, PIP_2_ or PIP_3_, suggesting that these denucleated cells may contain a PI pool which does not cycle through its phosphorylated forms in the same manner as C38:4 PIPn. In addition, the stimulation of the PI3K and PLC pathways in platelets indicated that some minor PIPn species present different dynamics to the C38:4 PIPn pool [108].

Alternative PIPn species might also be enriched in certain cell compartments. The exosomes released by prostate cancer cell line PC-3, showed a higher proportion of C34:1 and C36:1 species in PI compared with the parental cells [110]. As PI levels were very low in exosomes, their unusual composition could indicate an exclusion of polyunsaturated PI species during exosome formation. This lack of C38:4 enrichment in PIs has also been observed in extracellular vesicles derived from other prostate cell lines [111] or differentiated 3T3-L1 adipocytes [112], although their levels were not compared with those of the parental cells. Similarly, PI in lung surfactant showed a predominance of saturated/monounsaturated species, which could be related to its biophysical functions [25,113].

A higher saturation degree in alternative PIPn species could render functional advantages, such as facilitating their packing [3]. PIPn can aggregate in microdomains, as seen with clusters of synthasin-1A/PI(4,5)P_2_ controlling neuronal exocytosis [114]. Although these clusters could be spontaneously recreated in artificial membranes with both di-C18:1-PI(4,5)P_2_ and pig brain PI(4,5)P_2_ the influence of their acyl chain composition was not explored. Alternatively, the presence of more saturated, shorter chain species may simply reflect the lack of a requirement to remodel towards C38:4, because their main function might be distinct from their better established signalling roles e.g simply to provide more membrane.

While the enrichment in C38:4 species is generally preserved in primary cultures of mammalian cells, it is often lost in immortalized cell lines [115] (Figure 1D). The distribution of species in PIPn in these cells can be highly influenced by the fatty acid composition of the culture media, with C38:4 being dependent on the availability of C20:4 [9]. In fact, the distribution of PI species *in vivo* might be influenced by diet, as shown in obese rats fed with an insect-based chow, presenting a decreased proportion of C38:4 PI in the liver, though the total PUFA-PI was sustained by an increase in C38:3 PI [116]. Yet, different cell lines cultured in the same conditions can present extremely different proportions of C38:4 in PIPn. High levels of short and less saturated PI species have been observed in certain cancer cell lines [117], a phenotype that has also been observed in human tumours [118,119]. Mutations in p53 could be one of the factors favouring this unusual composition in PIs [120], possibly by altering the expression of genes involved in fatty acid synthesis [121]. Therefore, the systematic comparison of gene expression and mutation patterns in cell lines and tumours in relation to their % of C38:4 in PIPn could provide new insights into the mechanisms and functions behind this enrichment.

## Future perspectives

There are still some major gaps in our knowledge of how the acyl chain composition of PIPn are created in different organisms and, in particular, how enrichment for the C38:4 species occurs in most mammalian tissues. To some extent, this reflects the technical challenges in measuring these different species, particularly the less abundant, more highly phosphorylated PIPn. Hopefully, recent developments in MS methods will enable more routine measurements and, importantly, allow independent regio-isomers of PIPn to be interrogated (ie PI3P vs PI4P vs PI5P; PI(3,4)P_2_ vs PI(4,5)P_2_ vs PI(3,5)P_2_ [122]. Without this knowledge, it is very difficult to envisage selective interventions to directly test hypotheses regarding the function of acyl chain composition. However, it is also the case that the acyl chains of PIPn have been largely ignored in considering their interaction with effectors, and this should be more carefully addressed in future structural and biochemical studies, particularly where the acyl chains are directly engaged e.g. in LTPs. There has clearly been evolutionary pressure to engineer the acyl chain composition of PIPn to different degrees, in different tissues and organisms, we just do not at present have a satisfactory explanation for it.

**Perspectives**

- PIPns present an unusually uniform distribution of molecular species, with a marked predominance of the stearoyl/arachidonoyl hydrophobic backbone.
- This enrichment is achieved by the acyl chain remodelling of PI molecules involving selective acyltransferases and might provide molecular identity with the PIPn pool.
- New insights should arise from the study of the metabolic dynamics of PIPn molecular species and the inclusion of the acyl chain variable in functional and structural assays.

## Abbreviations

AGPAT: acylglycerol-3-phosphate acyltransferase
CDP-DG: CDP-diacylglycerol
CDS: CDP-diacylglycerol synthase
CL: cardiolipin
DG: diacylglycerol
DGK: diacylglycerol kinase
ER: endoplasmic reticulum
IRKs: inward-rectifier potassium channels
LPA: lysophosphatidic acid
LTPs: lipid transfer proteins
MEFs: mouse embryonic fibroblasts
MS: mass spectrometry
PIPn: Phosphoinositides
